# Validation of the Korean version of the Summated Xerostomia Inventory among older adults residing in nursing homes

**DOI:** 10.1186/s12889-024-18875-2

**Published:** 2024-05-31

**Authors:** SeolHwa Moon, Eunmi Oh, Daum Chung, Rina Choi, Gwi-Ryung Son Hong

**Affiliations:** 1 Department of Nursing, Hoseo University, #20, Hoseo-ro 79beon-gil, Baebang-eup, Asan, 31499 Korea; 2 Research Institute of Nursing Science, Hanyang University, #222 Wangsimni-ro, Seongdong-gu, Seoul, 04763 Korea; 3 College of Nursing, Hanyang University, #222 Wangsimni-ro, Seongdong-gu, Seoul, 04763 Korea

**Keywords:** Validity, Reliability, Xerostomia, Older adults, Nursing home

## Abstract

**Background:**

Xerostomia is commonly experienced by older individuals. We sought to develop and evaluate the reliability and validity of the Korean version of the Summated Xerostomia Inventory (K-SXI) among older adults residing in long-term care facilities (LTCFs) in Korea.

**Methods:**

In this secondary data analysis study using cross-sectional data, a cross-cultural adaptation process was conducted for the Korean version before data collection. Data collection was conducted from July 2021 to January 2022, targeting 544 older adults in 16 LTCFs. Data analysis included intraclass correlation coefficient (ICC) for test–retest reliability, and Cronbach’s α for internal consistency reliability. Exploratory and confirmatory factor analyses were used to verify construct and convergent validity. Test–retest analysis was performed 6 weeks after baseline. Convergent and concurrent validities were assessed with age group and the xerostomia standard single question, respectively.

**Results:**

A total of 544 older adults participated in this study. The mean of total K-SXI score was 11.70 (standard deviation, 4.96) points. The ICC value was calculated to be 0.90, and Cronbach’s α of K-SXI was 0.92. Exploratory factor analysis revealed a single factor, explaining 74.8% of the total variance, however, some goodness-of-fit indices of the single factor model were found to be unsuitable in confirmatory factor analysis. The convergent and concurrent validity were supported.

**Conclusion:**

The present study provides evidence supporting the validity and reliability of the K-SXI for measuring xerostomia in institutionalized older adults in Korea.

## Background

Xerostomia, defined as a subjectively determined dry mouth, is a common symptom experienced by 29.6–59.0% of older adults [1–3]. Symptoms can be caused by either systematic (e.g., endocrine disease, autoimmune disease, and infections) or local factors (e.g., medications, head and neck radiation, and lifestyle factors) [4]. Common chronic conditions of xerostomia affect speech, chewing, tasting, and swallowing, resulting in malnutrition and poor general health in the older population [5]. In addition, recent studies have reported that xerostomia is closely related to the deterioration of oral health–related quality of life in older adults [6].

Since older adults tend to take many medications for chronic diseases, drug-induced xerostomia is very common among them [7]. In a recent Italian population-based study, polypharmacy was more common among older adults residing in long-term care facilities (LTCFs) than those living in the community [8]. The presence of xerostomia among them is widespread [7]; thus, it is important to evaluate xerostomia for the management and prevention of xerostomia in older adults residing in LTCFs.

The diagnosis of xerostomia requires a comprehensive assessment, encompassing medical history-taking, oral examination, subjective dry mouth questions, and measurement of the salivary flow rate [4]. The measurement of salivary flow rate is commonly performed as a test for the diagnosis of hyposalivation, and it is usually measured for at least 5 min after fasting overnight or for more than 2 hrs to exclude other confounding factors [4]. The unstimulated whole salivary flow rate is evaluated with the patient in an upright sitting position, and salivary secretion is measured by continuously discharging saliva accumulated in the mouth into a prepared instrument [4]. Because of this cumbersome procedure to assess xerostomia, older adults residing in LTCFs who are cognitively and physically frail can have difficulty cooperating.

Given the significant impact of xerostomia on health outcomes in older adults, assessment of xerostomia should be easily performable by any healthcare professional [7]. Therefore, numerous questionnaires to assess subjective xerostomia have been developed to identify and assess the severity of xerostomia [5]. The Xerostomia Inventory (XI) is a reliable instrument for measuring dry mouth among older adults [5]. XI consists of 11 items and measures the severity of xerostomia in daily life [9]. Items cover experiential and behavioral aspects (eating, drinking, and swallowing) and dryness of the eyes and lips [9]. Although the XI translated into Korean was confirmed to have acceptable psychometric features, XI contains items covering dryness of the eyes, nose, or facial skin but not dry mouth directly, which limits its applicability to the older population [10]. Thomson and colleagues developed the five-item Summated XI (SXI), omitting some superfluous items, and determined its validity and properties [10]. The SXI consists of five items and can be used quickly and easily in clinical settings. It is suitable for frail older adults, and its validity and reliability have been accepted by targeting the older population in several countries [11–13].

Although different scales have been accepted as valid in other cultures, health-related scales require a process for cross-cultural adaptation to maintain the psychometric properties of the original scales due to socioeconomic, cultural, and linguistic diversity of the participants [30]. Accordingly, the purpose of this study was to translate SXI into Korean and then to evaluate the reliability and validity of the new Korean version of SXI (K-SXI) for older adults residing in LTCFs.

## Methods

### Study design and sample

The present study was a secondary analysis performed using data from the first year of a longitudinal study to identify the factors of mortality in LTCFs. The aim of this methodological study was (1) to translate the SXI developed by Thomson and colleagues [10] into Korean and then (2) to verify the validity and reliability of the K-SXI among older adults residing in LTCFs. We included patients who (1) were aged ≥ 65 years and residing in LTCFs, (2) were able to communicate, (3) were residing in an LTCF for > 2 months, (4) were able to understand the purpose of the study, and (5) agreed to participate in the study. Data collection was completed with 561 older adults in 16 LTCFs, but 544 participants were included in the analysis after excluding those with incomplete responses. The number of participants in this study met the required sample size of 150–200 for instrument verification [14]. In the final analysis, 544 participants were randomly assigned to either exploratory factor analysis (EFA) (*n* = 277) or confirmatory factor analysis (CFA) (*n* = 277) groups using SPSS 23.0 (IBM Corp., Armonk, NY, USA) software. The sample size for the test–retest reliability was 27 participants, considering that the number of participants per item was 5.37 [15].

### Data collection

Data collection was carried out from July 2021 to January 2022, and the survey was conducted through face-to-face interviews with participants by the staff of each LTCF. A manual was provided for the description of the measurement. General and clinical characteristics were collected from medical charts. The details of the data collection and training process of research assistants are described in a previous publication of this ongoing study [16]. At 6 weeks after baseline data collection, a test–retest analysis was conducted with 27 participants in one facility.

### Measurement

We collected general and clinical characteristics of age, sex, number of chronic diseases, and number of current medications.

The SXI consists of five simple and easy-to-answer items, and its validity and reliability were accepted for older adults residing in LTCFs in other countries [10, 17]. The K-SXI consists of five items and one standard question about xerostomia, as presented in Table 1. Participants were asked to respond to each item of the K-SXI on a 5-point Likert scale ranging from 1 (never) to 5 (always). The total score of the K-SXI, which is the sum of the five items, ranges from 5 to 25 points, and the higher the score, the greater the severity of dry mouth. To assess the concurrent validity of the K-SXI, the XI standard question “How often does your mouth feel dry?” was used [10], with the following response options: 1 = never, 2 = sometimes, 3 = often, and 4 = always.

**Table 1:** The original English and Korean versions (K-SXI) of the Summated Xerostomia Inventory (SXI)

Category	Summated Xerostomia Inventory in English	Summated Xerostomia Inventory in Korean (K-SXI)
Items	My mouth feels dry when eating a meal	음식을 먹을 때 입이 건조함을 느낍니다.
	My mouth feels dry	내 입안이 건조함을 느낍니다.
	I have difficulty eating dry foods	나는 마른 음식을 먹기가 힘듭니다.
	I have difficulties swallowing certain foods	나는 어떤 음식들은 삼키기가 힘듭니다.
	My lips feel dry	내 입술이 건조함을 느낍니다
Scoring	Never (1), almost never (2), sometimes (3), often (4), always (5)	전혀 그렇지 않다(1), 거의 그렇지 않다(2), 가끔 그렇다(3), 자주 그렇다(4), 항상 그렇다(5)
Standard question	How often does your mouth feel dry?	얼마나 자주 입안이 건조합니까?
Scoring	Never (1), sometimes (2), often (3), always (4)	전혀 그렇지 않다(1), 가끔 그렇다(2), 자주 그렇다(3), 항상 그렇다(4)

### Translation of the SXI into Korean and establishment of content validation

After obtaining approval from Dr. Thomson for the use of the instrument, a six-stage cross-adaptation process was carried out according to the guideline of Beaton and colleagues [18]. First, two bilingual translators translated the SXI from English into Korean. Both translations were synthesized after review by an expert in geriatric nursing. Second, a different translator conducted back-translation of the Korean version into English blinded to information about the original version. Third, after translation and back-translation of SXI, the content validity of the scale was verified using the item–content validity index by the experts. An expert panel consisting of five nursing professors performed the content and semantic evaluation of the original scale and translated version of the SXI. Each item was evaluated on a three-point scale (3 = ‘Exactly the same meaning in both versions,’ 2 = ‘Almost the same meaning in the two versions,’ 1 = ‘Different meaning in each version’) [19]. Three experts awarded 3 points to the four items; however, two experts awarded 2 points to item 4, so this item was modified to reflect their opinions. The original version of item 4, “I have difficulties swallowing certain foods,” was translated and back-translated as “I have difficulties swallowing some foods.” Reflecting the opinions of two experts, “some” was modified into “specific.” A pilot–test analysis was conducted with 10 older adults residing in LTCFs to assess the comprehension of the items. The final version of K-SXI was established without any additional modifications according to the pilot test (Table 1).

### Statistical analysis

Statistical analysis was conducted using SPSS 23.0 (IBM Corp., Armonk, NY, USA). Test–retest analysis was estimated using the intraclass coefficient (ICC), and the result was interpreted based on the following guideline [20]: less than 0.5 as poor; between 0.5 and 0.75 as moderate; between 0.75 and 0.90 as good; and greater than 0.90 as excellent. Internal consistency was determined with Cronbach’s α and item–total correlation (ITC). The construct validity was checked using EFA and CFA. To evaluate the suitability of EFA, Kaiser–Meyer–Olkin analysis and Bartlett’s test of sphericity were performed. The goodness-of-fit of the model was verified based on the following parameters: chi-square/degree of freedom (CMIN/DF) (≤ 3), normal fit index (NFI) (≥ 0.80), goodness-of-fit index (GFI) (≥ 0.80), adjusted GFI (AGFI) (≥ 0.80), Tucker–Lewis index (TLI) (≥ 0.80), comparative fit index (CFI) (≥ 0.80), root mean square error of approximation (RMSEA) (≤ 0.08), and standardized root mean residual (SRMR) (≤ 0.08) [21, 22]. The convergent validity was verified using the average variance extracted (AVE) (≥ 0.50) and construct reliability (CR) (≥ 0.70) [21, 22]. Additionally, we investigated the relationship between age and K-SXI score. An independent t-test was conducted to identify differences in K-SXI scores according to age group. The concurrent validity was confirmed by assessing the relationship between K-SXI scores and categories of xerostomia standard question using one way analysis of variance.

## Results

The characteristics of all 544 participants are shown in Table 2. The mean age was 83.64 (standard deviation [SD], 7.37) years, and 78.9% were female. The mean numbers of diagnosed diseases and number of current medications were 2.43 (SD, 1.06) and 8.17 (SD, 3.60), respectively. The total mean score of K-SXI was 11.70 (SD, 4.96) points, and each item score ranged from 2.23 to 2.43 (Table 3).

**Table 2:** Characteristics of the participants (*N* = 544)

Characteristics	Mean (SD)	*n* (%)
Age (years)	83.64 (7.37)	
Sex		
Male		115 (21.1)
Female		429 (78.9)
Number of diagnosed diseases	2.43 (1.06)	
Number of current medications	8.17 (3.60)	

**Table 3:** Item analysis and internal consistency of K-SXI (*N* = 544)

Item	Mean (SD)	Corrected ITC	Cronbach’s α if item deleted	Cronbach’s α
Item 1	2.23 (1.04)	0.860	0.785	
Item 2	2.39 (1.06)	0.848	0.785	
Item 3	2.43 (1.16)	0.834	0.780	
Item 4	2.30 (1.14)	0.837	0.781	
Item 5	2.36 (1.08)	0.796	0.789	
Total	11.70 (4.96)	1.00		0.918

Abbreviations: ITC, item–total correlation.

### Reliability

On the test–retest exam, the mean score of K-SXI was 10.30 (SD, 2.88) at baseline and 10.85 (SD, 2.14) after 6 weeks, resulting in an ICC of 0.904 (*P* < .001). The corrected ITC values of five items were all > 0.796, and Cronbach’s α of K-SXI was 0.918 (Table 3).

### Validity

**Fig. 1:**
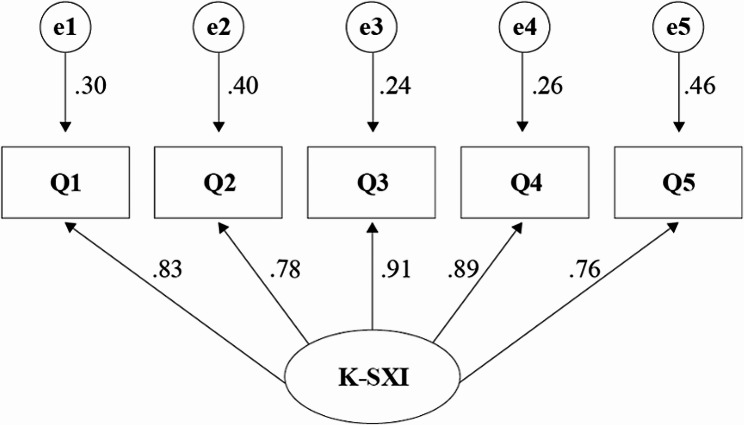
Standardized single-factor structural model of K-SXI.

The EFA and CFA were conducted to establish the construct validity. Before EFA, the results of Kaiser–Meyer–Olkin analysis (0.828) and Bartlett’s test for sphericity (*x*^*2*^ = 1026.46; *df* = 10; *P* < .001) were confirmed suitability of the data for EFA. One factor was extracted with an eigenvalue of 3.74 and total explained variance of 74.8%, and the factor loading of each item in single factor ranged from 0.70 to 0.81. Based on the results of EFA, a single-factor model of CFA was designed. The results of CFA are presented in Table 4; Fig. 1. The model of goodness-of-fit indices (*x*^2^ [*P*] = 133.85 [< 0.001], CMIN/DF = 26.77, NFI = 0.879, GFI = 0.811, AGFI = 0.433, TLI = 0.765, CFI = 0.883, RMSEA = 0.308, and RMR [SRMR] = 0.066 [0.061]) did not meet the standard suggested by the literature except for NFI, GFI, CFI, and RMR (SRMR) [22, 23].

**Table 4:** Goodness-of-fit indices for the confirmatory factor analyses of K-SXI

Model	CMIN/DF	NFI	GFI	AGFI	TLI	CFI	RMSEA	RMR (SRMR)
Initial model	26.77	0.879	0.811	0.433	0.765	0.883	0.308	0.066 (0.061)
Standard value	≤ 3	≥ 0.800	≥ 0.800	≥ 0.800	≥ 0.800	≥ 0.800	≤ 0.08	≤ 0.08

Abbreviations: AGFI, adjusted goodness of fit index; CFI, comparative fit index; CMIN/DF, chi-square/degree of freedom; GFI, goodness of fit index; NFI, normal fit index; RMSEA, root mean square error of approximation; SRMR, standardized root mean residual; TLI, Tucker–Lewis index.

**Fig. 2:**
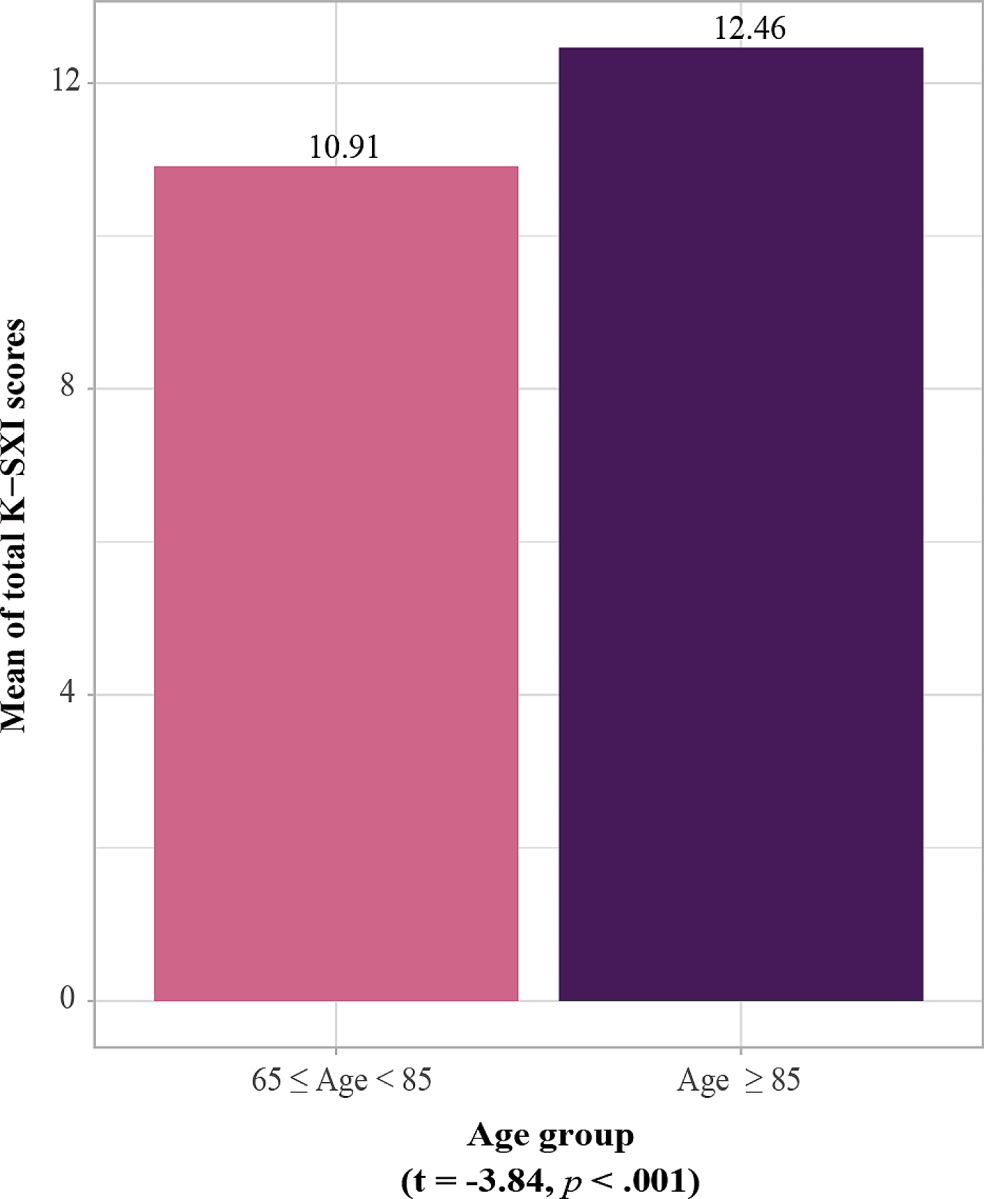
Mean K-SXI score by age groups

The values of convergent validity met the criteria (AVE = 0.86, CR = 0.91). We confirmed the distribution of mean of total K-SXI according to two age groups (65–84 years vs. 85 years and older) with statistically significant difference (*t* = -3.84, *p <* .001) (Fig. 2).

**Fig. 3:**
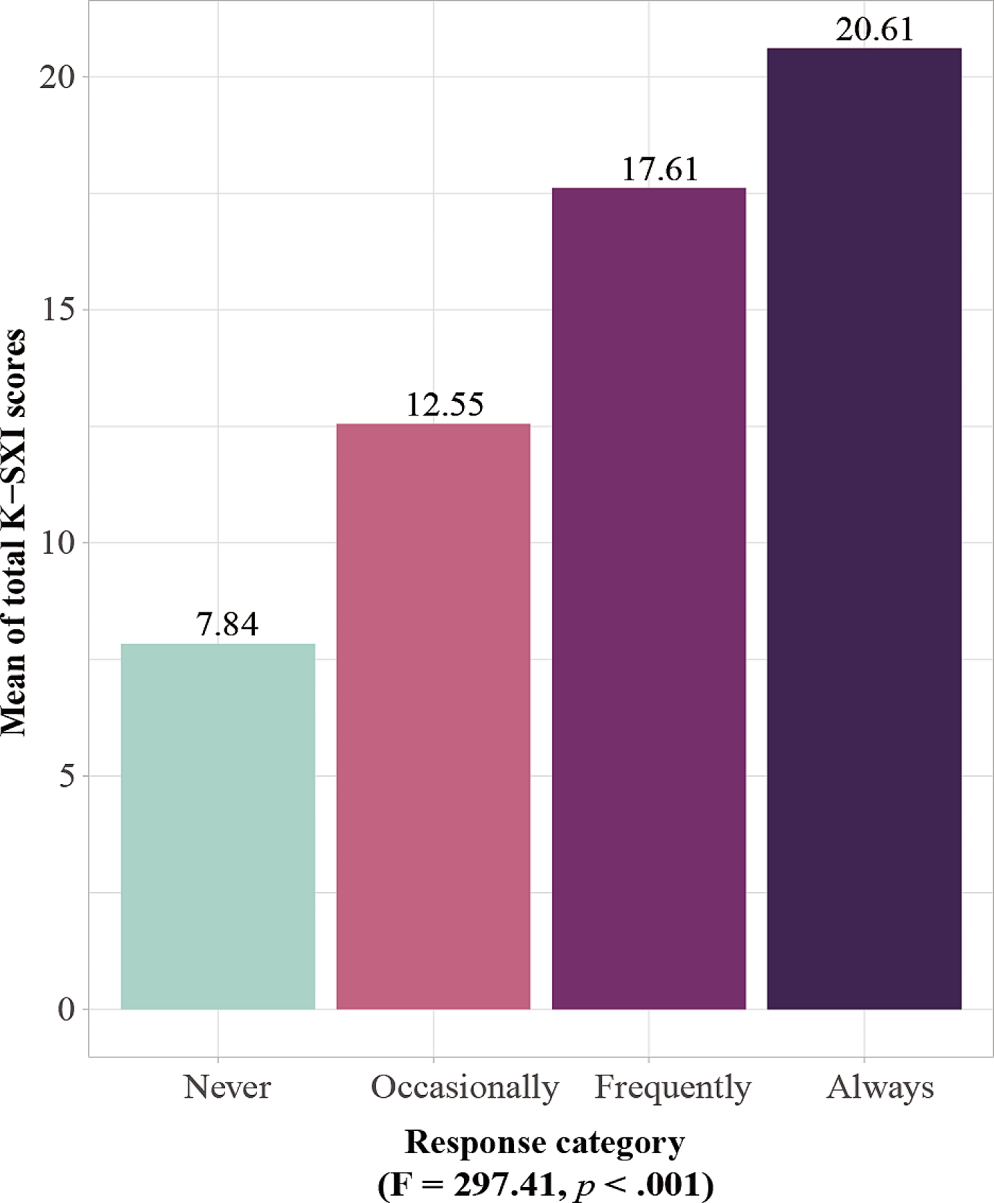
Mean K-SXI score by response category of xerostomia standard question

The mean of total K-SXI score was compared with four categories based on the xerostomia standard question to evaluate concurrent validity (Fig. 3). The mean K-SXI score was greatest in the group with severe dry mouth, and there was significant difference among the groups (F = 297.41, *p* < .001).

## Discussion

The present study evaluated the psychometric properties of the K-SXI with older adults residing in LTCFs. The results contended that K-SXI has good reliability and acceptable construct, convergent, and concurrent validities.

Test–retest analysis confirmed the reliability of the K-SXI by calculating the ICC and internal consistency with Cronbach α. During the data-collection period of the present study, it was difficult to contact potential participants and to secure cooperation from LTCFs under the coronavirus disease 2019 pandemic situation. Accordingly, the retest was performed 6 weeks after the baseline test, resulting in a delay of more than the optimal test–retest interval of 2–4 weeks in older adults [15]. Furthermore, the sample size for the retest did not meet the appropriate size of 30–50 individuals suggested in a previous study [24]. However, a systematic review [15] suggested that the sample size (ratio of the number of participants per item) of the test–retest analysis for older adults is 5.37:1. Despite the limitation of a longer interval before the retest, the ICC value was 0.90, which was confirmed to be an excellent degree of agreement [25]. The ICCs of SXI translation studies in other countries have ranged from 0.90 to 0.99 [17, 26]. The mean age of the participants in this study was 89 years old. That is not only higher than the participants in the previous study (aged 64 to 69 years) [17, 26], but despite the longer than optimal test-retest interval [15], similar ICC results were obtained compared to the previous study [17, 26]. This study conducted test-retests in only a small number of participants at one of 16 LTCFs, so these older adults might not fully reflect the cognitive or health characteristics of all participants.

The overall reliability with Cronbach’s α of the K-SXI in this study was 0.92, which was greater than the original SXI Cronbach’s α range of 0.70–0.80 [10]. The Cronbach α value represents the overall reliability of each item for the tool, and the results of this study satisfied the lower limit of acceptable values of 0.60–0.70 [27]. In the present study, the corrected ITC coefficient values for the five items were all ≥ 0.80. These results mean that K-SXI secured high internal consistency reliability. The findings of reliability testing support the K-SXI as a reliable and stable tool to use for older adults in LTCFs in Korea.

EFA was performed to evaluate the construct validity. K-SXI was confirmed to have a single-factor structure consistent with the original version [10] and translated versions from other countries, including China [12] and Turkey [26], and the total variance was considered satisfactory, at > 60% [21]. As the factor loading value of each item exceeded 0.70, it was confirmed that the items had good internal consistency to properly measure xerostomia [28]. The results of EFA in this study were consistent with translated versions of SXI, such as Chinese [12] and Turkish [26] versions. On the other hand, in the CFA, some goodness-of-fit indices of the single factor model were found to be unsuitable, which differed from the previous study [12]. According to the literature, such results could be influenced by the normality of data [29]. In the present study, while exploring the data for each item of SXI, normality was confirmed in histogram and skewness-kurtosis results, but the results of the Shapiro-Wilk test (*p* < .001) did not indicate a normal distribution. This may have been influenced by the measurement characteristics of each item (ordinary indicator: 5-point Likert scale). In addition, 27 staff members in 16 LTCFs participated in the data collection process, due to access restrictions related to the COVID-19 pandemic at the time of data collection [16]. Although a training procedure using video clip and manuals was performed to minimize the risk of inter-rater reliability, measurement errors might have occurred due to the multiple raters, and may have affected the results.

Both AVE and CR for the convergent validity, which indicates whether the items constituting the model adequately explain latent variables, met the criterion. This verified that all items of the K-SXI consistently explain xerostomia. Also, the mean K-SXI score according to the standard question response category increased with the frequency of xerostomia, and the result showed a positive slope. This was consistent with the results of the original and Portuguese versions of SXI [10, 11]. Mean of total K-SXI score was significantly different by age group, supporting the previous suggestion that aging is a major factor affecting xerostomia [4]. In addition, concurrent validity was evaluated using the xerostomia standard question proposed by Thomson and colleagues [10]. In the current study, the K-SXI score increased as the severity of dry mouth increased based on categories of xerostomia standard question; these results were similar to previous studies [10, 17].

### Limitations

The current study has some limitations. First, in this secondary data analysis, various factors related to xerostomia (e.g., medical history, oral condition, salivary flow rate, medication) [5] were not included in the validity analysis. As part of further investigations, we suggest confirming the psychometric properties of the K-SXI by including risk factors of xerostomia. Second, the participants of the current study could not be considered representative of all older adults in LTCFs in Korea. In this study, the test-retest period was extended to 6 weeks, and the study was conducted only in one LTCF. Depending on the type of LTCF, the level of cognition, health condition, and functioning of the older adults may have varied, and these characteristics might be reflected in the results. Therefore, future studies should apply an optimal test-retest interval to older adults in various types of LTCF. Also, we suggest testing K-SXI in a larger population of older adults residing in LTCFs. Despite these limitations, the present study is meaningful in that validity and reliability were confirmed by applying the self-reported K-SXI to older adults in LTCFs.

## Conclusion

The findings of this study suggest that the Korean version of the SXI has good psychometric properties and is a reliable and valid instrument for institutionalized older adults who are vulnerable to xerostomia.

## Data Availability

The datasets generated and/or analysed during the current study are not publicly available. The data cannot be shared publicly because of the contents contained within the ethics permission granted to this study from the Institutional Review Board of Hanyang University. Participants have not consented for their data to be provided to other researchers. But data are available from the corresponding author upon reasonable request.

## Abbreviations

LTCFs: long-term care facilities
K-SXI: Korean version of the Summated Xerostomia Inventory
XI: Xerostomia Inventory
EFA: Exploratory factor analysis
CFA: Confirmatory factor analysis
ICC: Intraclass coefficient
ITC: Item–total correlation
CMIN/DF: Chi-square/degree of freedom
NFI: Normal fit index
GFI: Goodness-of-fit index
AGFI: Adjusted GFI
TLI: Tucker–Lewis index
CFI: Comparative fit index
RMSEA: Root mean square error of approximation
SRMR: Standardized root mean residual
AVE: Average variance extracted
CR: Construct reliability
